# Alterations in the vaginal microbiota of patients with preterm premature rupture of membranes

**DOI:** 10.3389/fcimb.2022.858732

**Published:** 2022-08-08

**Authors:** Chunmei Yan, Fanzhen Hong, Gang Xin, Shuhong Duan, Xiaohui Deng, Yongping Xu

**Affiliations:** ^1^ Department of Obstetrics, The Second Hospital of Shandong University, Jinan, China; ^2^ Center for Reproductive Medicine, Department of Obstetrics and Gynecology, Qilu Hospital, Cheeloo College of Medicine, Shandong University, Jinan, China

**Keywords:** PPROM, PROM, vaginal microbiota, PTB, preterm birth

## Abstract

**Background:**

Preterm premature rupture of membranes (PPROM) is a common pregnancy complication. Yet, the main cause of PPROM remains poorly understood. In this study, we used 16S rRNA gene sequencing technology to identify the differences in vaginal microbiota between pregnant women with PPROM and those who delivered at term.

**Methods:**

Vaginal samples were collected from 48 patients with PPROM and 54 age- and gestational age-matched pregnant women who delivered at term (controls). The vaginal microbiota of the two groups was compared using 16S rRNA gene sequencing of the V3-V4 regions.

**Results:**

The vaginal microbial composition of the PPROM group was significantly different from that of the control group. Our results showed that the diversity of vaginal microbiota in patients with PPROM increased compared with controls. The relative abundance of *Lactobacillus iners, Gardnerella vaginalis, Prevotella bivia, Ochrobactrum* sp.*, Prevotella timonensis*, and *Ureaplasma parvum* were more abundant in patients with PPROM, while *Lactobacillus crispatus* and *Lactobacillus gasseri* were more abundant in controls. *Ochrobactrum* sp.*, Prevotella timonensis*, and *Gardnerella vaginalis*, could serve as biomarkers for PPROM. Finally, we proposed several metabolic pathways, including PWY-6339, PWY-6992, and PWY-7295.

**Conclusion:**

PPROM is characterized by vaginal microbial dysbiosis. The dysbiotic vaginal microbiota signatures in patients with PPROM include a higher bacterial diversity, decreased autochthonous bacteria, and increased pathogenic bacteria. These results may be beneficial for developing biomarkers for screening and early diagnosis of PPROM and may provide effective preventative treatments.

## Introduction

The premature rupture of membranes (PROM) is defined as membrane rupture prior to the onset of labor. PROM that occurs before 37 weeks of gestation is defined as preterm premature rupture of membranes (PPROM). PPROM increases neonatal and maternal disease risks, which is among the main causes of premature birth. PPROM leads to approximately 18%–20% of perinatal deaths in the United States. An increase in perinatal mortality and neonatal morbidity by 4-fold and 3-fold, respectively, has been observed in patients with PPROM. Common neonatal complications of PPROM include respiratory distress syndrome (RDS), intrauterine infection, and intraventricular hemorrhage (IVH). RDS occurs in 10%–40% of PPROM patients and leads to 40%–70% of neonatal deaths, while intrauterine infection occurs in 15%–30% of women with PPROM and causes 3%–20% of neonatal deaths (Dale et al., 1989; Yoon et al., 2000). Other neonatal complications include fetal pulmonary hypoplasia, skeletal deformities, cord prolapse, and neuro-developmental impairment (Yoon et al., 2000; Powell et al., 2021). In addition to neonatal complications, PPROM may also lead to complications in pregnant women. Intrauterine infection with clinical evidence has been observed in 15%–35% of women with PPROM, while postpartum infection occurs in approximately 15%–25% of PPROM cases (Garite and Freeman, 1982; Beydoun and Yasin, 1986; Kenyon et al., 2013). Moreover, placentae abruptio occurs in 2%–5% of pregnancies with PPROM (Ananth et al., 2004). These maternal and infant complications pose a serious burden on society and families.

Various pathological mechanisms, acting either alone or in combination with other factors, can lead to PPROM. For example, vaginal microbiota has an important role in the vagina (Turnbaugh et al., 2007) and can affect the health of the female genital tract (O'Hanlon et al., 2010). The abnormal vaginal microbiota is an important risk factor for PPROM (Coleman et al., 2013; Brown et al., 2018). Hypothetically, the colonization of pathogenic bacteria in the local and upper cervix and fetal membrane (Parry and Strauss, 1998) can lead to an inflammatory cascade reaction (Fortunato et al., 2002; Helmig et al., 2002; Shobokshi and Shaarawy, 2002), and in turn, the remodeling and destruction of membrane structure and premature rupture (Fortner et al., 2014). Previous studies on vaginal microorganisms have obtained data through microscopic examinations and traditional culture methods. However, owing to the large abundance and diversity of bacterial species in the vagina, not all bacteria may be identified through traditional culture, isolation, and identification methods (Reinhold-Hurek et al., 2015). Thus, information regarding vaginal microbiota is limited.

In recent years, the development of 16S rRNA gene sequencing technology has provided a new method for studying microbiota, but few studies have investigated the composition of vaginal microbiota in patients with PPROM (Baldwin et al., 2015; Kacerovsky et al., 2015; Paramel Jayaprakash et al., 2016). Studies on vaginal microbiota showed ethnic specificity between vaginal microbiota and preterm birth (Callahan et al., 2017). The integrated human microbiome program (iHMP) showed that the vaginal microbiota was a risk factor of preterm birth in African American women (Fettweis et al., 2019). Moreover, pregnant women of European descent with high levels of *Lactobacillus crispatus* showed a relatively low risk of preterm birth. Whilst the composition of vaginal microbiota differs between women of African and European descent, its impact on the risk of preterm birth remains controversial.

The relationship between the characteristics of vaginal microbiota in pregnant women in China and PPROM is still not well understood. Further research into the occurrence, prediction, and management of the pregnancy complications is required. In this study, we used 16S rRNA gene sequencing technology to compare the characteristics of vaginal microbiota between patients with PPROM and age- and gestational age-matched pregnant women who delivered at term to further investigate the relationship between vaginal microbiota and PPROM.

## Methods

### Study design and sample collection

The study was approved by the Second Hospital of Shandong University Research Ethics Board (KYLL-2018 (LW)043). All subjects provided written informed consent prior to sample collection.

This cross-sectional study aimed to compare the characteristics of vaginal microbiota between patients with PPROM and age- and gestational age-matched pregnant women who delivered at term (controls). All patients were enrolled at the Second Hospital of Shandong University between January 2019 and April 2020. The diagnosis of PROM included: (1) visual pooling of clear fluid in the posterior fornix of the vagina or leakage of fluid from the cervical os; (2) an alkaline pH of the cervicovaginal discharge (nitrazine test, pH ≥6.5), turning yellow nitrazin paper to blue; (3) microscopic fern-like crystals of the cervicovaginal discharge upon drying. The inclusion criteria for the PPROM group were: age of the pregnant women >18 years old, singleton gestation, gestational age between 24–36^+6^ weeks, and confirmed PROM based on clinical criteria ≤12 h. The exclusion criteria for the PPROM group were: the application of antibiotics, probiotics, and immunosuppressants in recent 2 months; fetal developmental abnormalities; complications, such as heart disease, immune diseases, cervical surgery, and so on. The inclusion criteria for the term delivery group included: age of pregnant women >18 years old, singleton gestation, normal routine examination of leucorrhea, and gestational age between 24–36^+6^ weeks. The exclusion criteria were: history of preterm labour or PPROM; with PPROM; application of antibiotics, probiotics, and immunosuppressants in recent 2 months; fetal developmental abnormalities; complications, such as heart disease, immune diseases, cervical surgery, and so on.

Three swabs were collected from the women with PPROM at enrolment: a cervical swab for microbial cultivation and two vaginal swabs from the anterior vaginal fornix, lateral and anterior vaginal wall (one for Nugent scoring and one for vaginal microbiota analysis). All swabs for the microbiota analysis were stored at -80°C within 2 h of sampling, and all swabs for cultivation and Nugent scoring were presented to the central laboratory of the Second Hospital of Shandong University. Vaginal secretions were cultured with a blood plate.

Demographic and clinical characteristics were collected from medical records and participants.

### DNA extraction and 16S rRNA gene sequencing

Bacterial genomic DNA from vaginal swabs was extracted using a modified cetyltrimethylammonium bromide (CTAB) protocol. The V3-V4 region of the 16S rRNA genes was amplified by PCR and sequencing was performed using the Illumina NovaSeq PE250 platform with NovaSeq 6000 SP Reagent Kit (500 cycles) at Shanghai Personal Biotechnology Co., Ltd (Shanghai, China).

### Bioinformatic analysis of 16S rRNA sequencing

The 16S rRNA sequencing data analysis was performed using Quantitative Insights Into Microbial Ecology (QIIME2 V. 2019.4) (Bolyen and Rideout, 2019) and R packages (v3.2.0). DADA2 software, wrapped in QIIME2, was used to quality filter, denoise, merge, and remove chimera (Callahan et al., 2016). Non-singleton amplicon sequence variants (ASVs) were aligned with Multiple Alignment using Fast Fourier Transformation (Katoh et al., 2002) and used to construct a phylogeny with FastTree2 (Price et al., 2009). The α-diversity metrics and β-diversity metrics were estimated using the diversity plugin with samples rarefied to 60308 sequences per sample (Lozupone and Knight, 2005; Lozupone et al., 2007). Taxonomy was assigned to ASVs using the classify-sklearn naïve Bayes taxonomy classifier in a feature-classifier plugin (Bokulich et al., 2018) against the Greengenes Database (DeSantis et al., 2006) (Release 13.8, http://greengenes.secondgenome.com/). Lactobacillus species were further annotated and validated by manual BLAST search in the National Biotechnology Information Center (NCBI) database.

### Statistical analysis

The α-diversity indices were calculated using the ASV table in QIIME2, visualised by R script as box plots to intuitively show the differences in diversity and richness between the PPROM group and term delivery controls. Statistical significance was verified using the Kruskal-Wallis rank-sum test and Dunn’s test *post-hoc*. A β-diversity analysis was performed to investigate the structural variation of microbial communities between the two groups using Jaccard metrics and was visualized using nonmetric multidimensional scaling (NMDS) and principal coordinate analysis (PCoA). Analysis of similarities (ANOSIM) using QIIME2 was used to assess whether the differences in microbiota structure between the two groups was significant. We used linear discriminant analysis effect size (LEfSe) analysis to identify taxa or pathways that were differentially abundant between patients with PPROM and term delivery controls (Segata et al., 2011). This method uses the non-parametric factorial Kruskal-Wallis rank-sum test to detect elements with significant differential abundances and linear discriminant analysis (LDA) to calculate the effect size of each. The operating characteristic curves (receiving operational curve, ROC) were constructed, and the area under the curve (AUC) was calculated to assess the diagnostic performance of the model using a random forests algorithm for analysis based on the species that were significantly different according to LEfSe analysis results. The correlation network was constructed using the SparCC algorithm python, and iGraph packages. The differences in maternal age and gestational age between the PPROM and term delivery groups were tested by an independent sample *t*-test using SPSS software. Other statistical analyses were performed, such as Fisher’s exact test. A P-value < 0.05 was considered to be statistically significant.

## Results

### Clinical characteristics of the study participants

Data were derived from vaginal microbiota of a large and well-characterized cohort which comprised 48 patients with PPROM and 54 age- and gestational age-matched pregnant women who delivered at term (controls). **Table 1** shows the detailed demographic and clinical features of all women involved in the study. Bacterial vaginosis is a mixed infection caused by vaginal dysbacteriosis, mainly characterized by the increase of thin vaginal secretions with a fishy smell. Abnormal vaginal secretion culture refers to the growth of aerobic bacteria on the culture plates.

**Table 1:** Clinical characteristics of the study subjects.

	PPROM group	Term delivery group	P value
Maternal age, years (mean±SD)	31.0±5.03	29.67±3.71	0.072
Gestational age at admission (mean±SD)	32.55±2.73	31.34±3.47	0.066
Racial/ethnic background (subjects)	all chinese	all chinese	
Vulvovaginal candidiasis (VVC)	1/48,2.1%	0/54	
Bacterial vaginitis(BV)	1/48,2.1%	0/54	
Vaginal secretion smear G + cocci	22/48,45.8%	–	
Vaginal secretion smear G-bacilli	5 /48,10.4%	–	
Vaginal secretion smear G + cocci/G-bacilli	8/48,16.7%	–	
abnormal vaginal secretion culture	9/48,18.8%	–	
abnormal leucorrhea routine (RT)	2/48,4.2%	0/54	
PH (mean±SD)	5.71±0.41	4.81±0.46	
Nugent Score (mean±SD)	3.56±1.24	2.95±0.99	
Group B streptococcal (GBS) carrier	2/48,4.2%	2/54	

P value, Comparisons between the PPROM group and term delivery group.

### Species accumulation curve

The species accumulation curve tended to be flat, indicating that the total number of ASV in the community would not significantly increase with new samples and that the sample size was sufficient (**Supplementary Figure S1**).

### Bacteria differentially abundant in PPROM versus controls

At the genus level, the most abundant vaginal bacteria in term delivery controls were *Lactobacillus* (relative abundance: 91.8% ± 23.7%; data shown as mean ± SD), followed by *Gardnerella* (relative abundance: 3.2% ± 13.1%; data shown as mean ± SD) and *Atopobium* (relative abundance: 2.9% ± 12.5%; data shown as mean ± SD). Most samples in the control group were dominated by *Lactobacillus* (90.74%), and few samples were dominated by *Gardnerella* (3.70%) and *Prevotella* (1.85%). The dominant taxa was defined as having a relative abundance of more than 50% (**Supplementary Figures S2A, B**). In the PPROM samples, the most abundant genus was *Lactobacillus* (relative abundance: 71.5% ± 32.6%; data shown as mean ± SD), followed by *Gardnerella* (relative abundance: 6.9% ± 7.1%; data shown as mean ± SD) and *Prevotella* (relative abundance: 4.4% ± 10.7%; data shown as mean ± SD). The majority of the PPROM group was dominated by *Lactobacillus* (77.1%) (**Supplementary Figures S2C, D**).

Compared to controls, the proportion of *Lactobacillus* as the dominant taxa decreased significantly, and the proportion of non-single genera as the dominant genus increased significantly (P<0.01, Fisher exact test).

**Figure 1:**
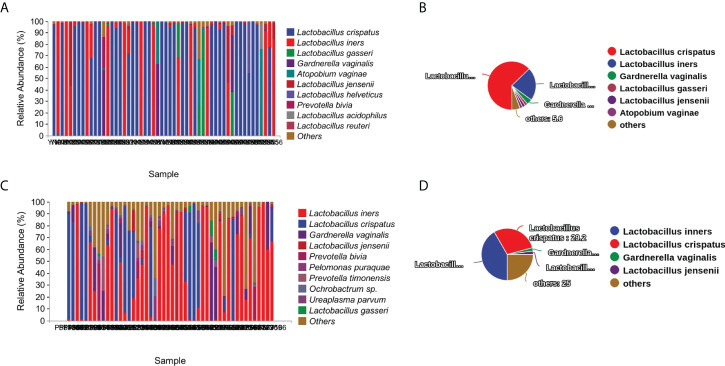
Vaginal microbiota composition and percentage of dominant taxa at species level. **(A)** Vaginal microbiota composition in the term delivery group at the species level. **(B)** Percentage of dominant species in the term delivery group. **(C)** Vaginal microbiota composition in the PPROM group at the species level. **(D)** Percentage of dominant species in the PPROM group.

The most abundant species found in the term delivery controls was *Lactobacillus crispatus* (relative abundance: 59.6% ± 46.8%; data shown as mean ± SD), followed by *Lactobacillus iners* (relative abundance: 24.2% ± 38.9%; data shown as mean ± SD), and *Lactobacillus gasseri* (relative abundance: 3.5% ± 13.9%; data shown as mean ± SD) (**Figure 1A**). *Lactobacillus crispatus* was found to be the dominant species in 63% of the term delivery control samples. Furthermore, *Lactobacillus iners* was dominant in 12 samples (22.2%), and *Gardnerella vaginalis* was the dominant species in two samples (3.7%) (**Figure 1B**).

In the PPROM group, *Lactobacillus iners* (relative abundance: 41.5% ± 39.6%; data shown as mean ± SD), followed by the *Lactobacillus crispatus* (relative abundance: 25.6% ± 38.1%; data shown as mean ± SD), and *Gardnerella vaginalis* (relative abundance: 6.9% ± 14.2%; data shown as mean ± SD) (**Figure 1C**) were the most abundant species. Less than half of the PPROM samples were dominated by *Lactobacillus iners* (41.7%), 29.2% were dominated by *Lactobacillus crispatus*, one sample was dominated by both *Gardnerella vaginalis* and *Lactobacillus jensenii* (2.1%), and 25.0% were not dominated by a single species (**Figure 1D**).

By comparing the proportion of dominant species in the two groups, we found that the proportion of *Lactobacillus crispatus* and *Lactobacillus gasseri* as the dominant taxa in group PPROM significantly decreased, while the proportion of *Lactobacillus inners* as the dominant taxa increased significantly compared with the term delivery control group (P<0.01, Fisher exact test).

### Increased α-diversity and altered overall microbial composition in patients with PPROM

**Figure 2:**
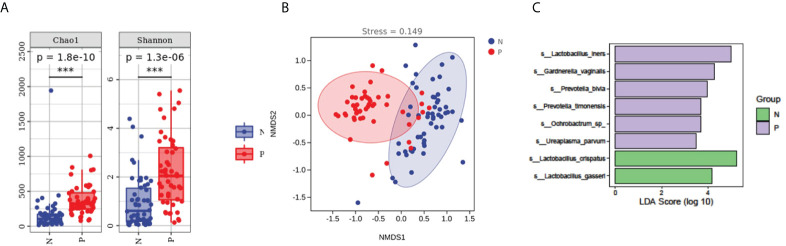
Comparisons of α- and β-diversities between the PPROM and term delivery groups. **(A)** Comparisons of α-diversity groups. ***It indicates that there is a significant difference between the two groups. **(B)** Comparisons of β-diversity between groups. **(C)** Differences in bacterial abundance in patients with PPROM versus controls.

To evaluate the diversity, abundance, and uniformity of the vaginal microbial communities, we compared the Chao1, Observed species, Shannon, Simpson, Faith’s PD, and Pielou’s evenness indices between the groups. Significantly higher diversity and abundance were observed in the vaginal microbiota from the PPROM group compared with the control group, as measured by Chao1 (p = 1.8e-10) and Shannon (p = 1.3e-6) diversity indices (Kruskal-Wallis rank-sum test, Dunn *post hoc* test) (**Figure 2A** and **Supplementary Figure S3A**).

We performed an NMDS analysis based on the Jaccard distance to assess the overall diversity in microbial composition. The results demonstrated a significant difference in vaginal microbial composition between the term delivery control and PPROM groups (p = 0.002; ANOSIM, stress = 0.0154; **Figure 2B**). PCoA based on Jaccard distance also demonstrated a significant difference between the two groups (pseudo-F: 6.53307, p = 0.002) (**Supplementary Figure S2B**).

### Difference in bacterial abundance in patients with PPROM versus controls

We performed LEfSe analysis on the vaginal microbiota composition of the two groups to identify which taxa were significantly different.

The relative abundance of *Lactobacillus iners, Gardnerella vaginalis, Prevotella bivia, Ochrobactrum sp, Prevotella timonensis*, and *Ureaplasma parvum* were significantly higher in women with PPROM, whilst the abundance of *Lactobacillus crispatus* and *Lactobacillus gasseri* were lower compared to the term delivery group (using LDA score=3.5) (**Figure 2C**).

### The vaginal microbiota-based signature associated with disease status

**Figure 3:**
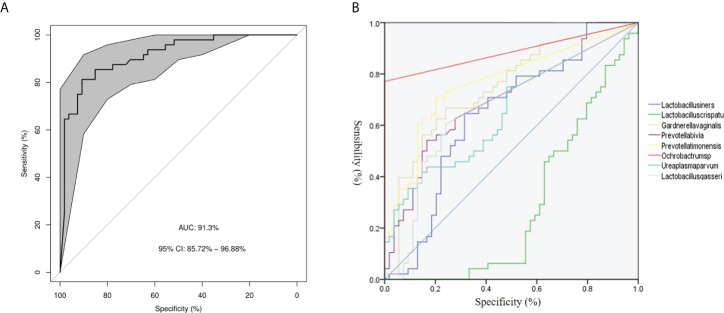
Disease classification based on vaginal microbiota signature. **(A)** The ROC curve of 8 different species. **(B)** The ROC curves of 8 different species.

We assessed the potential value of vaginal microbiota as a biomarker for predicting the PPROM risk.Using the random forests algorithm, we found that the combination of *Lactobacillus crispatus, Lactobacillus iners, Lactobacillus gasseri, Gardnerella vaginal, Prevotella bivia, Ochrobactrum* sp.*, Prevotella timonensis, and Ureaplasma parvum* could discriminate PPROM from controls, with an AUC of 91.3% (CI: 0.86–0.97) (**Figure 3A**). Additionally, using eight different species analyzed by LEfSe as predictors, and the generated AUC for ROCs (using SPSS 23), we found that *Ochrobactrum* sp. (AUC: 0.89, 95% CI: 0.81–0.96), *Prevotella timonensis* (AUC: 0.76, 95% CI: 0.67–0.86), and *Gardnerella vaginal* (AUC: 0.75, 95% CI: 0.65–0.84) could discriminate PPROM from controls (**Figure 3B**).

### Differential functions of bacteria in patients with PPROM versus controls

**Figure 4:**
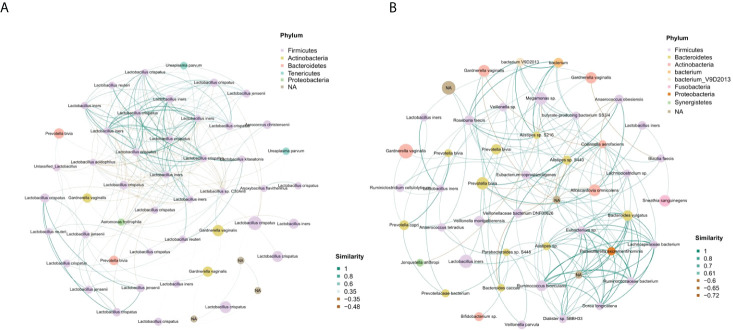
Identification of the vaginal microbial network. **(A)** The vaginal microbial network of the term delivery group. **(B)** The vaginal microbial network of the PPROM group.

We performed a bacterial community network analysis in both groups to explore the bacterial interactions of key species in the vaginal ecosystem. The vaginal microbiota from term delivery women showed that *Lactobacillus crispatus* and *Lactobacillus iners* were located at key positions in the microbiota network. Other *Lactobacillus* species, such as *Lactobacillus jensenii*, had a close interaction with *Lactobacillus crispatus* and *Lactobacillus iners* (**Figure 4A**).

In the PPROM group*, Lactobacillus crispatus* had no correlation with other species, and *Lactobacillus iners* formed a positive correlation function network with *Gardnerella vaginalis, Prevotella* sp.*, Veillonella montpellierensis* and *sneathia sanguinegens. Parasutterella excrementihominis* was in the key position and showed a positive correlation fuction network with *Bacteroides vulgatus*, *Eubacterium* sp. and *Ruminococcaceae bacterium* (**Figure 4B**).

**Figure 5:**
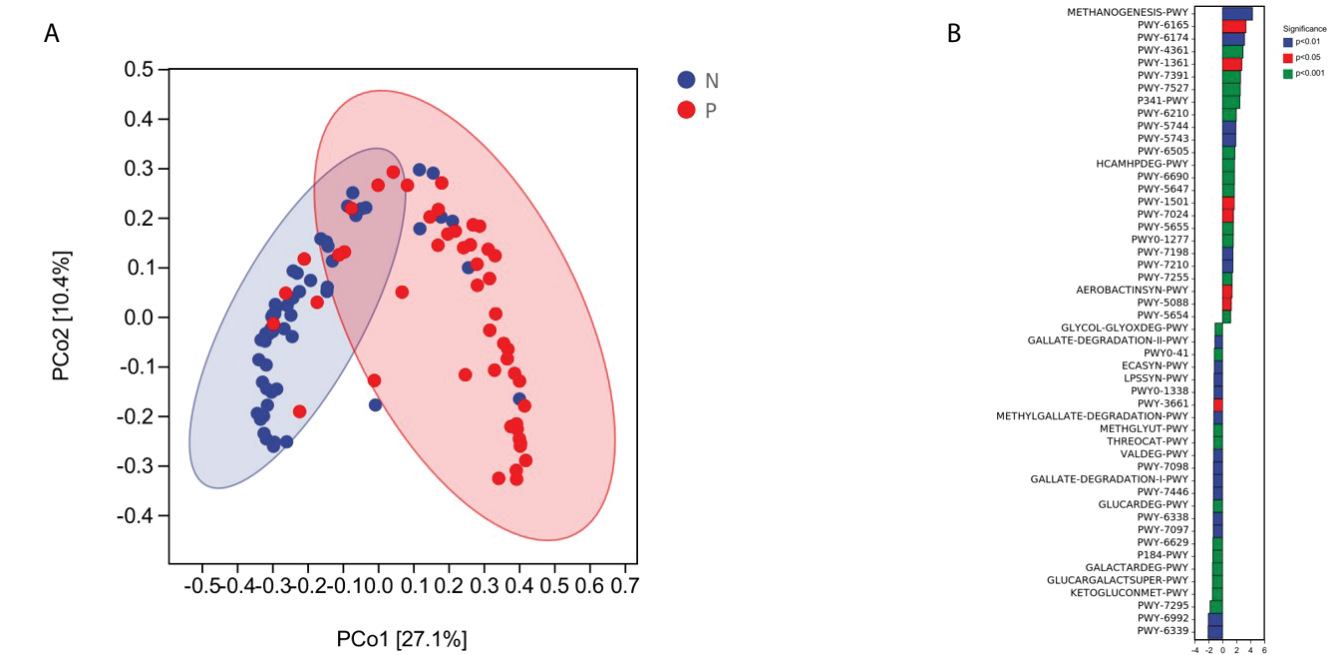
Comparisons of functions and metabolic pathways between the PPROM and term delivery groups. **(A)** Functional differences between groups were analyzed using principal coordinate analysis based on the Bray Curtis distance (p < 0.05). **(B)** Differential metabolic pathways identified by linear discriminant analysis effect size between groups.

Overall, the microbial function in women with PPROM was distinct from that of women in the term delivery group (**Figure 5A**). Moreover, 50 metabolic pathways were significantly different between the PPROM group and term delivery group (P <0.05), of which 23 were significantly upregulated in the PPROM group compared to the control group (P <0.05; **Figure 5A**). The most affected pathways included PWY-6339, followed by PWY-6992 and PWY-7295.

## Discussion

Previous studies have shown that vaginal microbiota is related to certain aspects of women’s health, such as preterm birth (Stout et al., 2017; Brown et al., 2019a). Studies conducted in Europe (Kindinger et al., 2017; Stafford et al., 2017) and North America (Callahan et al., 2017; Fettweis et al., 2019) on Caucasian populations have revealed positive associations between increased vaginal microbial diversity and risk of preterm birth; *Gardnerella vaginalis*, *Sneathia* spp.*, Prevotella* spp., and *Mollicutes* spp. were identified as microbial markers and risk factors. Moreover, studies in the United States investigating African decent women reported decreased vaginal diversity as a risk factor for preterm birth (Nelson et al., 2016). Recent publications have also demonstrated a low *Lactobacillus* spp. dominance and high species diversity in vaginal microbial communities in women with PPROM (Brown et al., 2019b).

In this study, we used 16S rRNA gene sequencing to further explore the difference in vaginal microbiota in Chinese women with PPROM and in term delivery. We found high diversity and low *Lactobacillus* dominance in the PPROM group, which is consistent with previous studies.

The vaginal microbiota fluctuates according to circulating hormone levels during a woman’s lifespan. The increased estrogen level promotes the proliferation of vaginal epithelial cells and the deposition of glycogen, and the thickened vaginal epithelium produces lactic acid (Linhares et al., 2011), resulting in the decrease of vaginal pH value, which is suitable for the colonization of acidophilic bacteria, such as *Lactobacillus* spp. *Lactobacillus spp* metabolizes abundant glycogen in the vagina producing lactic acid isomers, further reducing the vaginal pH value. Very few other microorganisms could survive in such an acidic environment. Therefore, *Lactobacillus spp* becomes the dominant commensal in most individuals (Hickey et al., 2012). Furthermore, during pregnancy, the placenta secretes a large amount of estrogen, which reduces the diversity of vaginal microbiota and promotes *Lactobacillus spp* dominance (Siimes et al., 1983). *Lactobacillus spp* dominance is established through competitive exclusion and secretion of some chemicals, such as bacteriocin and biosurfactants. Such environment protects the host against the colonization of pathogens. Previous studies have shown that these pathogens are related to bacterial vaginosis, pelvic inflammatory diseases, vulvovaginal candidiasis, sexually transmitted diseases, human immunodeficiency virus infection, and high-risk human papillomavirus infection (Velraeds et al., 1998; Borgdorff et al., 2014; Zheng et al., 2015). Our study demonstrated that the reduced *Lactobacillus spp* abundance and *Lactobacillus spp* dominance are related to the colonization of pathogenic bacteria, which may cause PPROM.

We further studied the species of *Lactobacillus* spp. Compared with term delivery women, *Lactobacillus iners* relative abundance and dominance increased in the patients with PPROM, while the abundance and proportion of *L. crispatus* dominant bacteria decreased. Kacerovsky *et al.* studied the cervical microbiota of 61 patients with PPROM using hierarchical clustering methods and found 4 community state types (CSTS) of cervical microbiota: CSTI (*L. crispatus* dominance), CST III (*L. iners* dominance), CST IV-A (*non-Lactobacillus* dominance) and CST IV-B (*G. vaginalis* and *sneathia sanguinegens* dominance). *Non-Lactobacillus* CST type was associated with a strong cervicitis reaction and a high incidence rate of microbial invasion of the amniotic cavity, while CSTI was associated with the lowest level of inflammation and microbial amniotic cavity invasion. Our study showed that 41.7% of the PPROM samples were dominated by *Lactobacillus iners*, 29.2% of the PPROM samples were dominated by *Lactobacillus crispatus*, 2.1% of the PPROM samples were dominated by *Gardnerella vaginalis*, and 25.0% were not dominated by a single species, which corresponds respectively to CST III, CST I, CST IV-B, and CST IV-A.

We further studied the relationship between microorganisms of the vaginal microbiota. *Lactobacillus crispatus* and *Lactobacillus iners* were located at the key positions in the microbiota network of the term delivery group. Other *Lactobacillus* species, such as *Lactobacillus jensenii*, closely interacted with *Lactobacillus crispatus* and *Lactobacillus iners.* In the PPROM group, *Lactobacillus crispatus* did not correlate with other species, and *Lactobacillus iners* formed a positive correlation network with *Gardnerella vaginalis, Prevotella* sp.*, Veillonella montpellierensis*, and *sneathia sanguinegens.* Therefore, the decrease of *Lactobacillus crispatus* dominance and the increase of *Lactobacillus iners* dominance may be one of the reasons for PPROM. We speculate that this may be caused due to the following reasons: due to the lack of gene encoding D-lactate dehydrogenase, *Lactobacillus iners* produces L-lactate and cannot maintain the acidity in the vagina, so it cannot provide sufficient protection to prevent vaginal dysbacteriosis (Saito et al., 2014). Compared with L-lactic acid, D-lactic acid has a stronger defense against potential pathogenic bacteria.

We also identified eight species that were significantly different in the vaginal microbiota of the two groups. Among them, decreased *Lactobacillus crispatus* and increased *Lactobacillus iners* were most related to the disease status, and the correlation between *Ochrobactrum spp* and PPROM has not been reported, which may be related to the ethnic specificity of the vaginal microbiota. ROC analysis of these eight differential species was further carried out to explore their clinical application. *Ochrobactrum* sp.*, Prevotella timonensis*, and *G. vaginalis* can be used as biomarkers to distinguish the disease status of PPROM. The discovery of these biomarkers provides a theoretical foundation for predicting, preventing, and treating PPROM. The vaginal microbiota of pregnant women can be screened in the early and middle trimesters of pregnancy. If the above biomarkers are positive and the abundance of *Lactobacillus crispatus* decreases, patients can be regarded as a high-risk group and thus can be treated accordingly.

We screened eight metabolic pathways, including the super pathway of methylglyoxal degradation (METHGLYUT-PWY), nicotinamide adenine dinucleotide biosynthesis pathway II (NADSYN-PWY), L-tryptophan biosynthesis super pathway (PWY-6629), L-tryptophan degradation pathway (PWY-5655), L-valine degradation pathway I (VALDEG-PWY), polymyxin resistance pathway (PWY0-1338), L-valine degradation pathway I (VALDEG-PWY), and Enterobacter common antigen synthesis pathway (ECASYN-PWY), which are related to infection. We hypothesized that these pathways are related to the pathogenesis of PPROM; yet, this hypothesis needs to be confirmed by further experiments. The super pathway of methylglyoxal degradation of vaginal microbiota increased in patients with PPROM, which was involved in the degradation of methylglyoxal. Methylglyoxal is a dicarbonyl compound, which is a by-product of glycolysis by some bacteria. Studies have shown that neutrophils produce methylglyoxal as an antibacterial agent during bacterial infection, and the infection of group A streptococcus is related to the degradation of methylglyoxal. Therefore, we infer that the methylglyoxal degradation super pathway may participate in the occurrence of PPROM (Zhang et al., 2016). The nicotinamide adenine dinucleotide biosynthesis pathway II of vaginal microbiota in the PPROM group decreased. Nicotinamide is an important molecule found in all living cells. The regulation of NAD metabolism has been proved to be related to a variety of diseases, including cancer, neurodegenerative diseases, and inflammatory diseases. Mesquita et al. showed that its regulation is also related to host-pathogen interaction (Mesquita et al., 2016). Other studies also showed that NAD could inhibit Candida infection (Belenky et al., 2007). In the PPROM group, the L-tryptophan biosynthesis super pathway increased, and the L-tryptophan degradation pathway decreased. Studies showed that abnormal metabolism of L-tryptophan may be related to abnormal pregnancy and infection (Badawy et al., 2016). Another study demonstrated that tryptophan metabolism was related to urogenital Chlamydia infection (Bommana et al., 2021). The L-valine degradation pathway I increased in the vaginal microbiota of patients with PPROM. Studies showed that L-valine increased the phagocytosis of macrophages and effectively killed Gram-negative bacteria (*Escherichia coli* and *Pseudomonas aeruginosa*) and Gram-positive bacteria (*Staphylococcus aureus*) (Chen et al., 2017). The occurrence of PPROM may be related to L-valine degradation. The polymyxin resistance pathway in vaginal microbiota of patients with PPROM increased. Polymyxin (PMX) is a cationic peptide antibiotic that binds to *Escherichia coli* lipopolysaccharide (LPS) and prevents inflammatory activation. Studies showed that PMX was associated with partial remission of intrauterine inflammation (Saito et al., 2014). At the same time, the Enterobacter common antigen synthesis pathway (ECASYN-PWY) in vaginal microbiota of patients with PPROM increased. Studies fund that Enterobacter antigen is associated with urogenital infection during pregnancy (Naumann et al., 1979). To sum up, in this study, the metabolic pathway of vaginal microbiota of patients with PPROM significantly changed compared with pregnant women of term delivery group, and the changed metabolic pathways were mostly related to infection, suggesting that infection has an important role in the pathogenesis of PPROM.

The present study has several limitations. First, this is a cross-sectional study that lacks information on the dynamic changes of the vaginal microbiota in patients with PPROM. Furthermore, although significant differences between the PPROM group and term delivery controls were identified, samples from the PPROM group were taken after membrane rupture. Thus, the microbiota characteristics could be affected by other substances, such as bacteria on the membrane and amniotic fluid. However, multiple studies have shown that amniotic fluid is very similar to a sterile environment (Sacks et al., 2018). Moreover, this study showed that the average vaginal pH value of patients with PPROM was in the normal range, suggesting that PROM and amniotic fluid outflow had little effect on vaginal pH value of PPROM pregnant women. So the influence of amniotic fluid should be limited. The PPROM is difficult to predict, so it is difficult to obtain vaginal microbial samples immediately before PPROM. In some studies, vaginal microorganisms were sampled several times during pregnancy to observe the difference in vaginal microbiota between pregnant women with PPROM and normal pregnant women. Because the occurrence of PPROM is difficult to predict, the sampling at fixed time points may be far away from the occurrence of PPROM, and the vaginal microbiota may change greatly during this period. Therefore, the state of vaginal microbiota at the time of sampling may be difficult to reflect the situation of vaginal microbial composition at the time of PPROM, and there may be great variation in the results. At the same time, the incidence rate of PPROM is 2%-4%. In order to obtain a considerable number of samples of patients with PPROM, thousands of samples need to be studied, which increases the cost. This study collected vaginal microbial samples within 12 hours of PPROM and before applying antibiotics. Since this field is still in its infancy and there is little data on the vaginal microbiota of patients with PPROM in the Chinese population, we believe our study provides valuable information to the field.

## Conclusion

This study characterized the changes in vaginal microbiota in women of Chinese nationality, comparing patients with PPROM with age- and gestational age-matched pregnant women who delivered at term. We identified several taxa that significantly increased in the vaginal microbiota in patients with PPROM, where altered signalling pathways were mainly related to infection. Our study provides valuable information on the correlation between vaginal microbiota and PPROM. Yet, further studies are required to determine this relationship’s underlying mechanisms and causality. Nevertheless, our findings provide valuable information to the field, concentrating on Chinese women with PPROM.

## Data availability statement

The datasets presented in this study can be found in online repositories. The raw data has been uploaded to NCBI (Accession to cite for these SRA data: PRJNA792044).

## Ethics statement

The studies involving human participants were reviewed and approved by Second Hospital of Shandong University, Jinan, Shandong. The patients/participants provided their written informed consent to participate in this study.

## Author contributions

XD and YX are co-correspondence authors and contributed equally. XD designed, supervised the study and wrote the manuscript. YX performed the analysis and provided constructive discussions. CY as the first author performed the study, helped analyze the data, and contributed to writing the manuscript. FH contributed to data analyses and helped write the manuscript. GX and SD collected and prepared samples and helped write the manuscript. All authors have read and approved the final manuscript.

## Funding

The project is supported by Yongping Xu Scientific Research and Development Fund (26010232007168).

## Conflict of interest

The authors declare that the research was conducted in the absence of any commercial or financial relationships that could be construed as a potential conflict of interest.

## Publisher’s note

All claims expressed in this article are solely those of the authors and do not necessarily represent those of their affiliated organizations, or those of the publisher, the editors and the reviewers. Any product that may be evaluated in this article, or claim that may be made by its manufacturer, is not guaranteed or endorsed by the publisher.

## Supplementary material

Supplementary Figure 1 Species accumulation curve.

Supplementary Figure 2 Vaginal microbiota composition and percentage of dominant taxa at the genus level. **(A)** Vaginal microbiota composition in the term delivery group at the genus level. **(B)** Percentage of the dominant genera in the term delivery group. **(C)** Vaginal microbiota composition in the PPROM group at the genus level. **(D)** Percentage of the dominant genera in the PPROM group.

Supplementary Figure 3 Comparisons of α- and β-diversities between the PPROM and term delivery groups. **(A)** Comparisons of α-diversity between groups. **(B)** PCoA analysis based on Jaccard distance between groups.
