# Supplementary figures and images for: Alterations in the vaginal microbiota of patients with preterm premature rupture of membranes
Source: Front Cell Infect Microbiol. 2022 Aug 8;12:858732. doi: 10.3389/fcimb.2022.858732 (PMC9393476; doi:10.3389/fcimb.2022.858732)

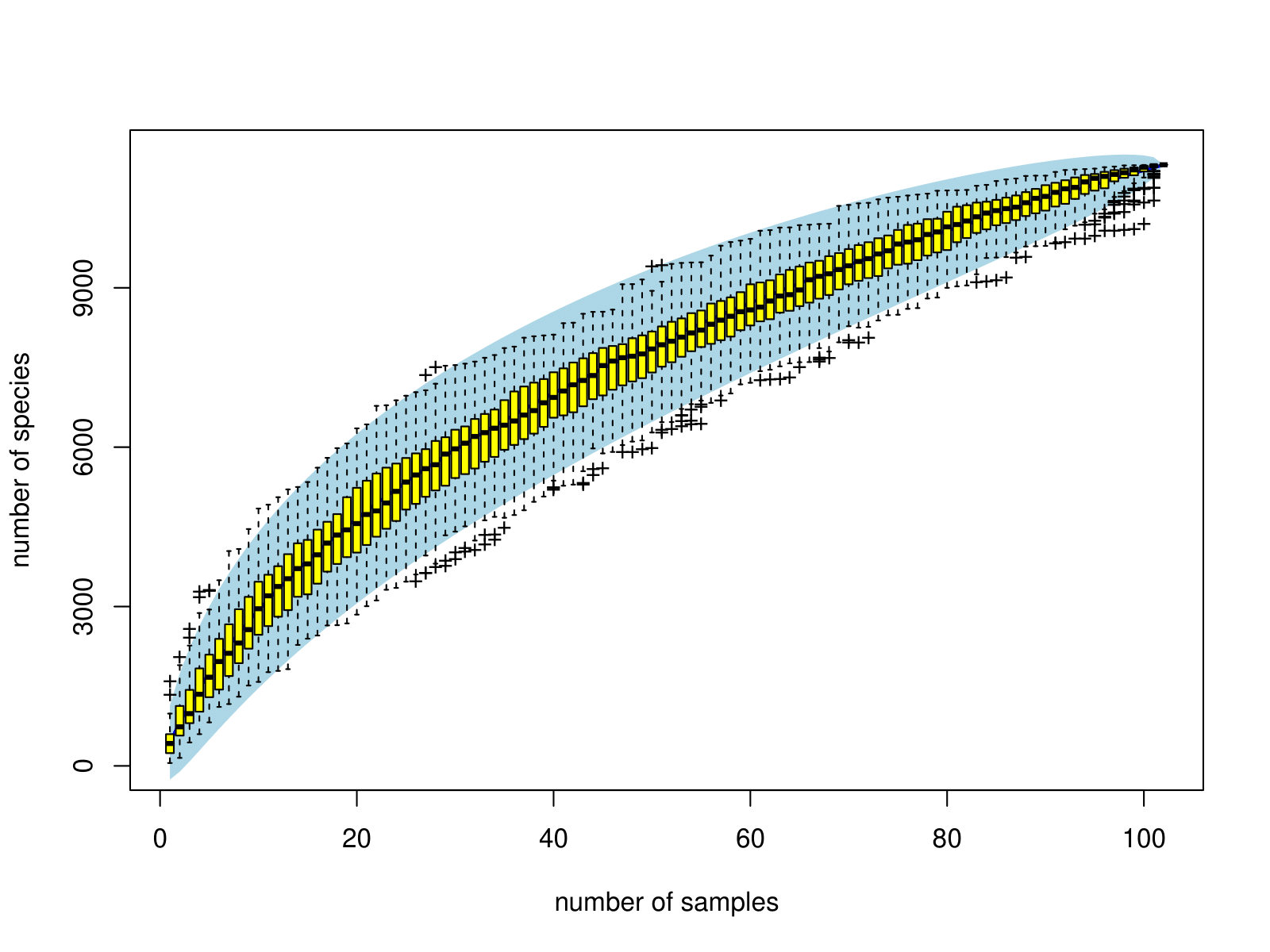

Supplement: Supplementary Figure 1 — Species accumulation curve. [file Image_1.png]

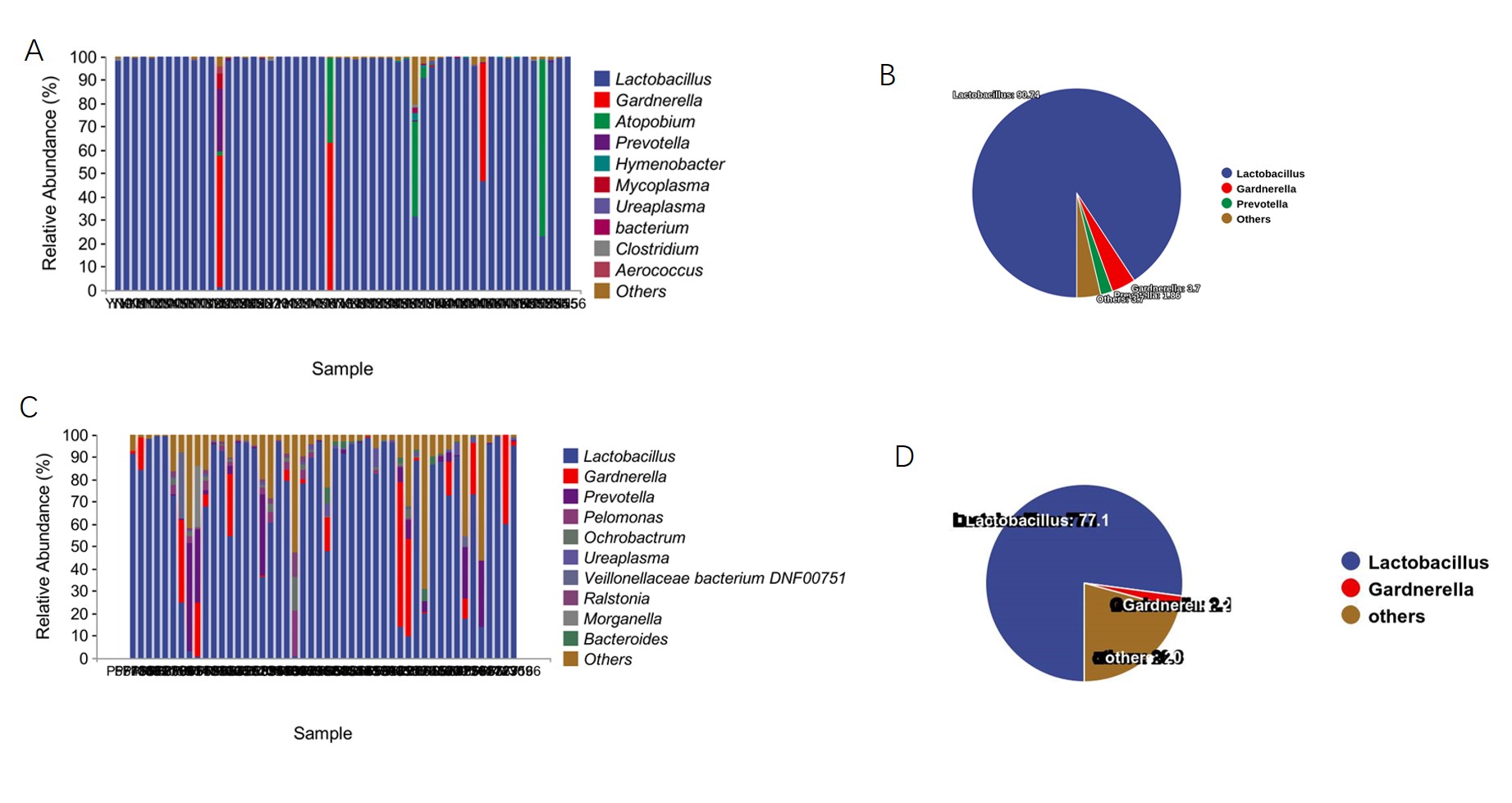

Supplement: Supplementary Figure 2 — Vaginal microbiota composition and percentage of dominant taxa at the genus level. (A) Vaginal microbiota composition in the term delivery group at the genus level. (B) Percentage of the dominant genera in the term delivery group. (C) Vaginal microbiota composition in the PPROM group at the genus level. (D) Percentage of the dominant genera in the PPROM group. [file Image_2.jpeg]

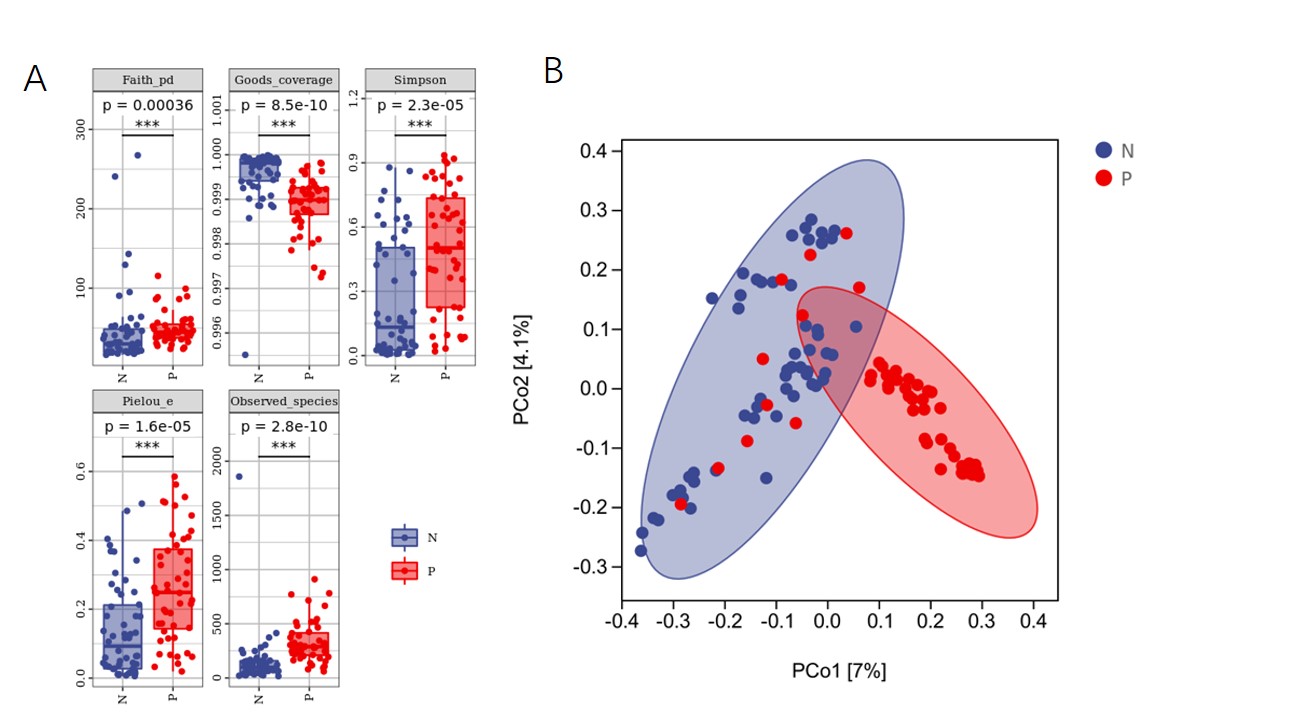

Supplement: Supplementary Figure 3 — Comparisons of α- and β-diversities between the PPROM and term delivery groups. (A) Comparisons of α-diversity between groups. (B) PCoA analysis based on Jaccard distance between groups. [file Image_3.jpeg]
